# Automated Early Detection of Myelodysplastic Syndrome within the General Population Using the Research Parameters of Beckman–Coulter DxH 800 Hematology Analyzer

**DOI:** 10.3390/cancers13030389

**Published:** 2021-01-21

**Authors:** Noémie Ravalet, Amélie Foucault, Frédéric Picou, Martin Gombert, Emmanuel Renoult, Julien Lejeune, Nicolas Vallet, Sébastien Lachot, Emmanuelle Rault, Emmanuel Gyan, Marie C. Bene, Olivier Herault

**Affiliations:** 1 Department of Biological Hematology, Tours University Hospital, 37000 Tours, France; noemie.ravalet@univ-tours.fr (N.R.); amelie.foucault@univ-tours.fr (A.F.); f.picou@chu-tours.fr (F.P.); m.gombert@hopitaux-drome-nord.fr (M.G.); e.renoult@chu-tours.fr (E.R.); s.lachot@chu-tours.fr (S.L.); e.rault@chu-tours.fr (E.R.); 2 CNRS ERL 7001 LNOX, EA7501, Faculty of Medidine, Tours University, 37000 Tours, France; emmanuel.gyan@univ-tours.fr; 3 Department of Hematology and Cell Therapy, Tours University Hospital, 37000 Tours, France; julien.lejeune@chu-tours.fr (J.L.); nicolas.vallet@inserm.fr (N.V.); 4 Department of Biological Hematology, Nantes University Hospital, 44000 Nantes, France; marie-christine.bene@chu-nantes.fr

**Keywords:** myelodysplastic syndrome, cell blood count (CBC), blood smear

## Abstract

**Simple Summary:**

A substantial fraction of the elderly population suffers from moderate anemia, and blood smear analysis can guide towards a diagnosis of myelodysplastic syndrome (MDS). Nevertheless, in medical laboratories, blood smear review is only performed when quantitative or qualitative flags occur upon complete blood count (CBC). Consequently, the suspicion of MDS can be delayed in the absence of systematic blood smear observation, which is crucial to initiate a full diagnosis process by cytological analysis of bone marrow aspiration. The Beckman Coulter DxH 800 hematology analyzer (Beckman-Coulter, Brea, CA) is widely used over the world. We propose in this study the clinical use of 10 unexploited “research parameters” for early detection of subclinical MDS by selective triggering of blood smear examination.

**Abstract:**

The incidence of myelodysplastic syndrome increases with aging and the early diagnosis enables optimal care of these diseases. The DxH 800 hematology analyzer measures and calculates 126 cytological parameters, but only 23 are used for routine CBC assessment. The goal of this study was to use the 103 unexploited “research parameters” to develop an algorithm allowing for an early detection of subclinical MDS patients by triggering morphological analysis. Blood sample parameters from 101 MDS patients and 88 healthy volunteers were analyzed to identify the critical “research parameters” with: (i) the most significant differences between MDS patients and healthy volunteers, (ii) the best contributions to principal component analysis (PCA), first axis, and (iii) the best correlations with PCA, first two axes (cos^2^ > 0.6). Ten critical “research parameters” of white blood cells were identified, allowing for the calculation of an MDS-likelihood score (MDS-LS), based on logistic regression. Automatic calculation of the MDS-LS is easily implementable on the middleware system of the DxH 800 to generate a flag for blood smear review, and possibly early detection of MDS patients in the general population.

## 1. Introduction

Myelodysplastic syndromes (MDS) are a heterogeneous group of clonal hematopoietic disorders, characterized by hypercellular and dysplastic bone marrow yielding ineffective hematopoiesis and increased apoptosis, leading to cytopenia (anemia in most cases, as well as thrombocytopenia and/or neutropenia) [1]. The median age at onset is around 72–75 years old, and clinical presentation is dependent on the depth and type of cytopenia. Progression to acute myeloid leukemia (AML) is observed in about 30% of MDS cases. MDS is suspected upon detection of cytopenia and morphological changes of peripheral blood cells, and the diagnosis/prognosis must be comforted by bone marrow aspiration for morphologic, flow cytometry, cytogenetic, and when needed, molecular analyses [2,3]. In most cases, the indication to perform bone marrow aspiration is unambiguous. Nevertheless, when cytopenia is moderate in asymptomatic patients, the absence of blood smear examination can delay diagnosis [4,5]. Indeed, the alarm thresholds proposed by the manufacturers of complete blood count instruments are lower than normal CBC values, and blood smears are only reviewed upon generation of a flag triggered by the detection of cytopenia below these thresholds. Since CBC is one of the most frequent biological analyzes, automated hematology analyzers could be optimized to flag potential disorders, such as subclinical MDS.

The development of an original automated strategy to identify early morphological signs of MDS is of medical interest. The Beckman Coulter DxH 800 hematology analyzer measures and calculates 126 cytological parameters based on photometry, impedance, and light scatter. These parameters aggregate 23 conventional CBC parameters and 103 unused “research parameters”, including cell population data (CPD) and research-use-only (RUO) parameters. Light scatter data reflect the white blood cells’ (WBC) size, granularity, nuclear structure, and cellular complexity [6]. Therefore, their mean (MN) and standard deviation (SD) are affected by cellular morphological abnormalities. Several studies have reported that the DxH 800 “research parameters” could be of interest to predict leukemia and its lineage [7] or to detect infectious diseases, such as malaria [8], sepsis [9,10,11,12], or viral infection in children [13].

The aim of this study was to build an algorithm based on the unused “research parameters” of the DxH 800, allowing the calculation from “extended” CBC (conventional and research parameters) of an MDS-likelihood score (MDS-LS) that could allow for early detection of subclinical cases of MDS and trigger blood smear and cytological analysis. This algorithm is of clinical interest to potentially initiate early and efficient clinical management of MDS patients.

## 2. Results

### 2.1. Univariate and Multivariate Analyzes

**Figure 1:**
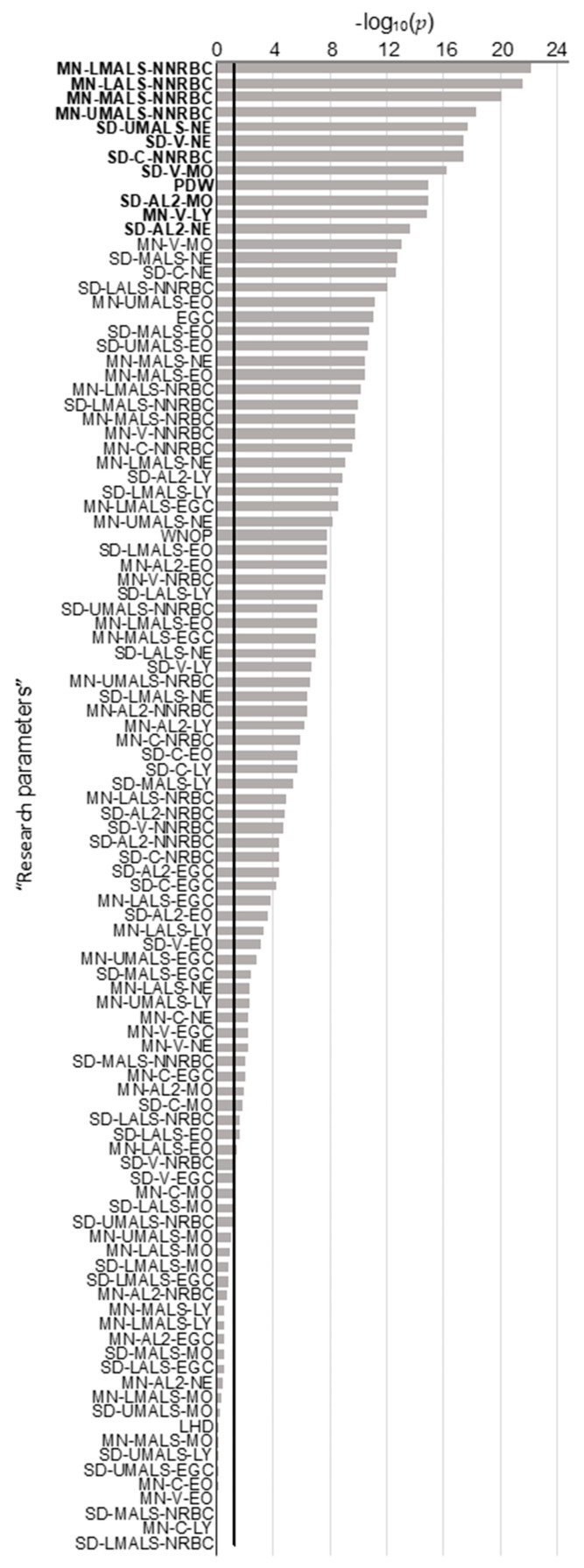
*p*-Values of monoparametric comparisons of each “research parameter” from myelodysplastic syndromes (MDS) patients and healthy volunteers. Parameters with values above the 5% threshold (black line y = −log10(0.05)) are significantly different between MDS patients and healthy volunteers. MN: Mean; SD: Standard deviation; LMALS: Low Median Angle Light Scatter; NNRBC: Non-Nucleated Red Blood Cells; LALS: Low Angle Light Scatter; MALS: Median Angle Light Scatter; UMALS: Upper Median Angle Light Scatter; NE: Neutrophils; V: Volume, MO: Monocytes; PDW: Platelet Distribution Width; AL2: Axial Light Loss; C: Conductivity; EGC: Early Granulated Cells; LY: Lymphocytes; WNOP: WBC estimate (corrected) from the NRBC Optical Channel, EO: Eosinophils; NRBC: Nucleated Red Blood Cells, LHD: Low Hemoglobin Density.

A large number of significant differences were observed between MDS patients and healthy volunteers in monoparametric analyses when considering all 126 CBC parameters (Figure 1 and Supplementary Tables S1 and S2). The cytopenias of MDS patients were mostly characterized by a significant decrease in hemoglobin (93.8 ± 18.1 g/L vs. 140.2 ± 9.2 g/L, *p* = 2.2 × 10^−29^) and platelet counts (154.7 ± 143.8 × 10^9^/L vs. 252.9 ± 48.8 × 10^9^/L, *p* = 1.6 × 10^−12^). Significant differences were also noted for WBC (4.7 ± 3.8 × 10^9^/L vs. 5.8 ± 1.2 × 10^9^/L, *p* = 7.8 × 10^−6^) and absolute neutrophil (2.9 ± 2.9 × 10^9^/L vs. 3.2 ± 0.8 × 10^9^/L, *p* = 3.9 × 10^−3^) counts. Conversely, a significant increase was observed in MDS patients for the mean corpuscular volume (but still within normal range (99.1 ± 12.3 fL vs. 92.2 ± 4.4 fL, *p* = 1.6 × 10^−5^)), mean platelet volume (*p* = 4.3 × 10^−6^), and red blood cell distribution (*p* = 1.9 × 10^−26^). Seventy-five unused “research parameters” were statistically significantly different (Figure 1 and Supplementary Table S2). Among them, neutrophil volumes (MN-V-NE and SD-V-NE, respectively) were significantly increased in MDS patients (*p* = 6.0 × 10^−3^ and *p* = 3.0 × 10^−18^). Conversely, decreased values were observed regarding neutrophil granularity in MDS patients (MN-MALS-NE, MN-UMALS-NE, and MN-LMALS-NE, *p* = 3.0 × 10^−11^, *p* = 5.7 × 10^−9^ and *p* = 6.4 × 10^−10^, respectively), indicating the expected hypogranularity of granulocytes.

**Figure 2:**
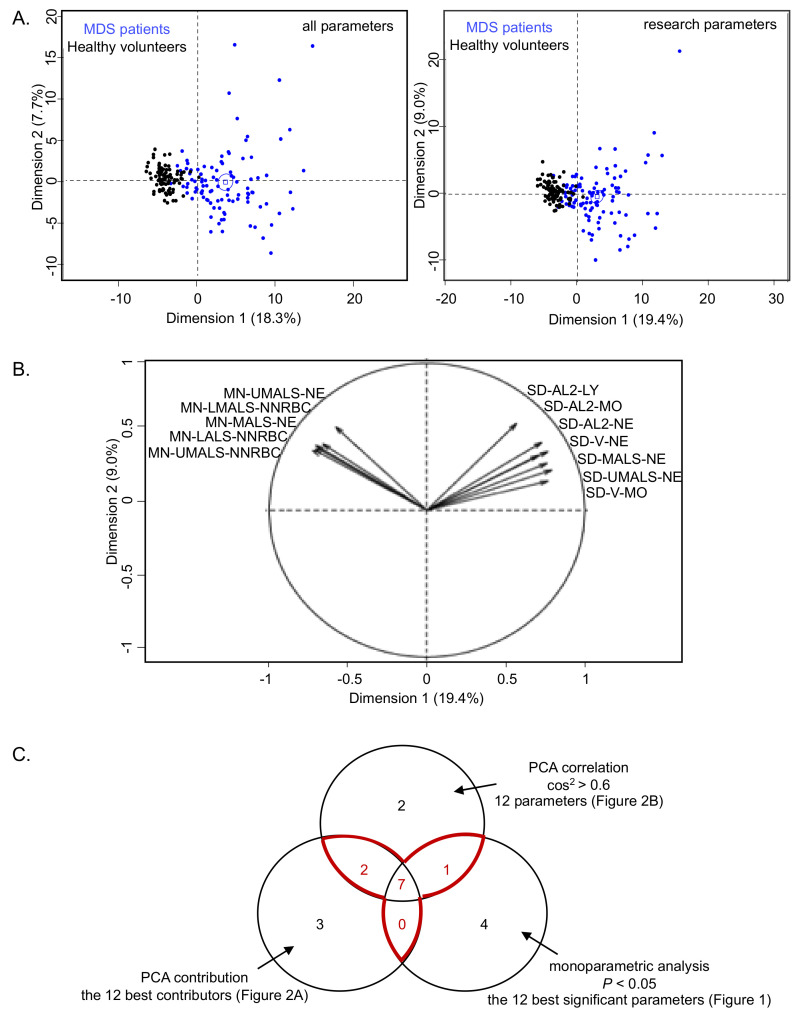
Principal component analyses (PCA) of MDS patients (blue) vs. healthy volunteers (black). (**A**) Individual graphs of PCA with all 126 parameters of the DxH 800 hematology analyzer (left panel) or with the 103 “research parameters” (right panel). Both PCAs show a satisfying segregation between the two groups. (**B**) Variables graph. Parameters with cos^2^ > 0.6 are illustrated. A high cos^2^ indicates a good representation of the parameter on PCA. The means of five CPD parameters of neutrophils and leukocytes are correlated negatively to the first axis. Conversely, standard deviation of 7 CPD parameters are correlated positively to the first axis. All parameters are related to leukocyte (NNRBC) and neutrophil characteristics, related to decreased granularity and cellular heterogeneity. (**C**) Venn diagram. Ten parameters of interest are identified by comparing MDS patients and healthy volunteers with monoparametric analysis, PCA contribution, and PCA correlation.

Since 75/102 “research parameters” were significantly modified in the MDS group, a multivariate analysis was performed. The combination of these approaches allowed for the identification of a limited and manageable core of 10 parameters of interest, avoiding potential bias due to a single statistical approach. PCA showed an efficient segregation of the groups of MDS patients and healthy donors (Figure 2A). Considering “research parameters”, Dimensions 1 and 2 represented 19.4% and 9.0% of the variability, respectively. The 12 parameters with the best correlations at the two first axes of PCA (cos^2^ > 0.6) are shown in Figure 2B. It is noteworthy that all are related to leukocytes (NNRBC) and mostly neutrophil characteristics, reflecting decreased granularity and cellular heterogeneity. This is in agreement with the well-known cytological abnormalities associated to MDS [14].

In order to optimize the use of these parameters of interest, a mathematical strategy was applied to develop the MDS-LS, and thus create a new MDS flag.

### 2.2. Mathematical Model Construction and Cross-Validation

Three lists of the 12 best parameters were established from (i) monoparametric analysis (Figure 1), (ii) PCA contribution (Figure 2A), and (iii) PCA correlations (Figure 2B). Ten parameters were common to at least two of these three lists (Figure 2C), all related to leukocytes: MN-LALS-NNRBC, MN-LMALS-NNRBC, MN-UMALS-NNRBC, SD-AL2-MO, SD-AL2-NE, SD-MALS-NE, SD-UMALS-NE, SD-V-MO, and SD-V-NE.

**Figure 3:**
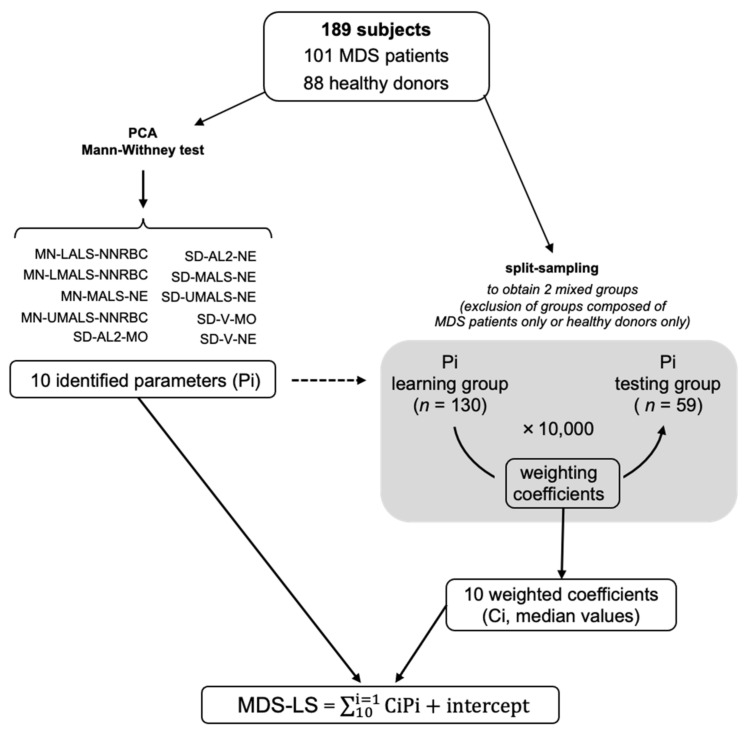
Mathematical model construction. Schematic representation of randomization to “learning” and “testing” groups for cross-validation.

During the split sample strategy (Figure 3), the ten common parameters selected were weighted by coefficients using logistic regression (Figure S1). This allowed for the calculation of an MDS-LS value for each sample as follows:

$$\mathrm{MDS}-\mathrm{LS}\;=\;\sum_{10}^{i=1}\mathrm{CiPi}+\mathrm{intercept},$$

where Ci = median of the weighting coefficient of each parameter i Pi = parameter i value. Ten thousand iterations were performed with a mean efficiency of 92.2 ± 6.4 × 10^−4^%, demonstrating the robustness of the strategy to identify MDS patients.

**Figure 4:**
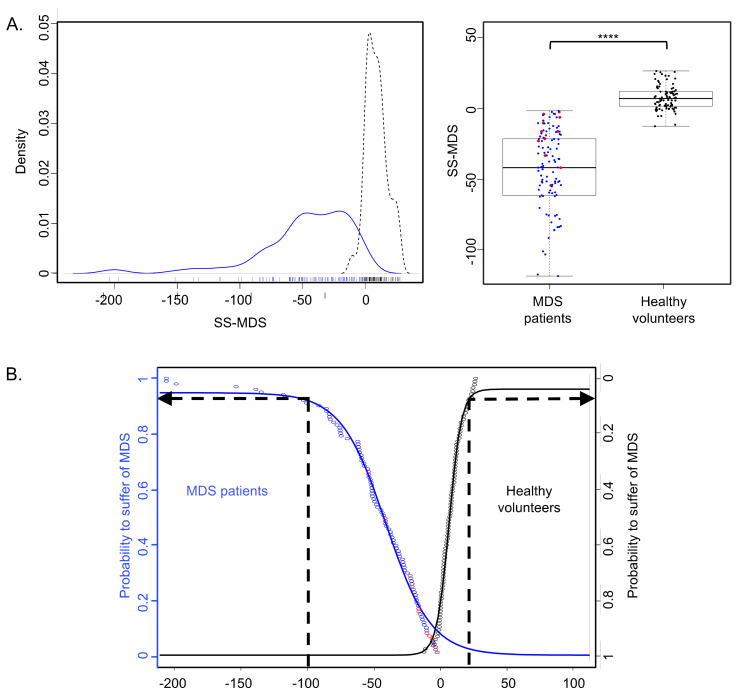
MDS likelihood score (MDS-LS) of MDS patients (blue) and healthy volunteers (black). In red, the 11 MDS patients for whom the MDS-LS would have been decisional to trigger a blood smear (no blood smear with the classical procedure). (**A**) MDS-LS density (left panel) or boxplots (right panel) of MDS patients and healthy volunteers. The outliers are not shown in the boxplot representation. MDS-LS of both cohorts are significantly different (****: *p* < 0.0001). (**B**) Empirical cumulative distribution function. The vertical axis represents the probability to suffer from MDS. If the MDS-LS is negative, there is an increasing probability of MDS, justifying blood smear. For example, if the MDS-LS value is −100, this probability is 95.4%.

The distribution of MDS-LS values was characterized by a narrow peak for healthy volunteers, while that of MDS patients was more heterogeneous (Figure 4A left). The score was significantly lower and always negative for MDS patients (−49.1 ± 38.4 vs. +7.6 ± 8.4, *p* < 2.10^−16^; Figure 4A right). With a threshold set to zero, the sensitivity and specificity of MDS-LS were 100% and 80.7%, respectively. The positive and negative predictive values were 93.5% and 100%, respectively.

In order to help the calculation of MDS probability (y), the empirical cumulative distribution function (Figure 4B) was determined as follows:

y = 0.957/(1 +e((−13.11 − [SS-MDS value])/(−15))).

For example, for an MDS-LS value of −100, the probability of suffering from MDS is 95.4%. Among the 101 MDS patients, the standard strategy of CBC determination failed to yield a blood smear in 11 cases. Interestingly, the MDS-LS was <0 in all of these cases, and would have triggered a blood smear review. Clinical and biological characteristics of these patients are detailed in Table 1. Of note, four of them had received red blood cell concentrate transfusions, and thus failed to trigger the “anemia” flag.

### 2.3. Independent Testing of MDS-LS

MDS-LS allowed us to suspect MDS for 92% (*n* = 23) of the 25 CBC coming from 22 MDS-diagnosed patients. As expected, the overall mean MDS-LS of these patients was negative (−58.8 ± 41.6). Two MDS patients (#17 and #18) were not detected (Supplementary Table S3). BM examination of patient #17 (MDS-SLD) showed isolated and moderate dyserythropoiesis in a context of chronic alcoholism, with a loss of chromosome Y in 76% of cells. Patient #18 (MDS-MLD) had dyserythropoiesis and dysgranulopoiesis with an isolated del(20q). An independent external cohort of 25 age-matched healthy controls (without hematological disease) allowed for the calculation of specificity. MDS-LS yielded a specificity of 84.0%, not different from that calculated from the first cohort (80.7%). These results are detailed in Supplementary Table S3.

## 3. Discussion

In this study, 10 CBC parameters were identified and combined in an MDS likelihood score or MDS-LS, potentially helpful to suspect MDS. These 10 parameters were identified by comparing 101 MDS patients and 88 age-matched healthy volunteers. They were selected from 103 “research parameters” of the DxH 800 instrument, by mono- and multi-parametric statistical analyzes. The MDS-LS was computed by logistic regression and internal cross-validation. More than 9 times out of 10, this new score allowed for efficient classification of subjects in subgroups of MDS patients or heathy volunteers, with good specificity and sensitivity. The interest of this strategy was verified by the ability of the MDS-LS to identify 11/101 MDS patients undetectable with the pre-configured CBC flags. These 11 patients presented too moderate anemia or thrombocytopenia to trigger the observation of a blood smear (threshold at 80 g/L and 100 × 10^9^/L, respectively). These situations especially occurred when patients transfused, deceiving the “low hemoglobin” flag (4 patients out of the 11 not detected).

Previous studies with various hematology analyzers have reported specific variations in extended CBC parameters for MDS patients [15]. Three generations of Beckman Coulter hematology analyzers (LH 750, LH 780, and DxH 800) have been tested, with increasing numbers of extended CBC parameters, especially regarding light scatter values. By using the LH 750 analyzer, Miguel et al. showed a decreased mean neutrophil light scatter and MN-C-NE in MDS patients [16], and Haschke–Becher et al. identified SD-C-NE as the most predictive parameter of MDS [17]. With the LH 780 analyzer, Raess et al. [18] found that platelet distribution width (PDW), standard deviation of red cell distribution width (RDW-SD), MN-C-NE, MN-V-NE, and SD-V-NE were the most discriminating RUO and CPD parameters. Of note, the latter were also identified in our study as significantly different between MDS patients and healthy volunteers. Two recent publications suggest that the DxH 800 analyzer could be of interest for MDS detection. The first one investigated extended CBC parameters for 37 MDS patients compared to 56 patients suffering from a myeloproliferative neoplasm (MPN), among which 11 had chronic myelogenous leukemia [19]. These authors identified a subset of 13 research parameters allowing for discrimination between MDS and MPN patients using ROC curve analyses for each parameter. The second study compared 43 MDS patients to 21 patients suffering from lymphoproliferative disorders (13/21) or presenting non-malignant anemia, leukocytosis, and erythrocytosis (8/21). Their results show that a combination of four research parameters could be of interest to identify MDS patients. These two studies led us to consider that research parameters of the DxH 800 could help in the early detection of MDS in the general population. The most important characteristics of our strategy were (i) a large series of 101 MDS patients, (ii) compared to a cohort of 88 truly healthy volunteers (as confirmed by through exploration, including bone marrow aspiration), (iii) a combined monoparametric and multiparametric approach, and (iv) calculation of the MDS-LS by logistic regression. This very stringent strategy allowed for the identification of 10 critical research parameters. Interestingly, six and three of them, respectively, had been reported in the two studies described above, comparing MDS to non-MDS patients [19,20].

With the ADVIA 2120 analyzer from Siemens Healthcare Diagnostics^®^ (Siemens Healthcare Diagnostics, Deerfield, IL, USA), Rocco et al. [21] published in 2011 a study that included 197 MDS patients. These authors identified 17 parameters combined in five different MDS-specific patterns. Several concerned neutrophils, in accordance with our results with the DxH 800 analyzer.

With Sysmex^®^ instruments (Sysmex^®^ Corporation, Kobe, Japan), Le Roux et al. [15] identified in 2010 the structural neutrophil parameter (NEUT-X) of the XE-2100 analyzer as interesting to identify MDS patients. More recently, on the XN-10 analyzer (Sysmex^®^ Corporation, Kobe, Japan), Boutault et al. [22] established a score which discriminates MDS patients from individuals with other causes of cytopenia. These authors identified three parameters to create this score: two from standard CBC (mean corpuscular volume and absolute neutrophil count), and one research parameter reflecting neutrophil complexity (Ne-WX). In our study, although we compared MDS patients to healthy controls, it is interesting to note that we obtained consistent results which highlight the abnormal diffraction of neutrophils in MDS. In addition, only leukocyte parameters emerged for the MDS-LS score calculation, even in anemic patients with MDS-SLD. For all of our 17 cases of MDS-SLD (with RS or not), dysplasia concerned the erythroid lineage. For nine of them, CBC was performed at diagnosis and yielded an MDS-LS lower than zero, suggesting that leukocytes were impacted by dysplasia, but not enough to be detected by cytological analysis, or not considered significant by the cytologist. It cannot be excluded that the algorithm calculating the MDS-LS may trigger the spread of smears in diseases other than MDS, but morphologic examination will be useful in such cases to clarify potential anomalies.

## 4. Materials and Methods

### 4.1. Cohorts Description

Two patient cohorts were used to perform this study. The first one allowed us to build the MDS-LS, which was validated with the second one.

The first cohort compared the results of the 126 CBC parameters measured by the Beckman–Coulter DxH 800 hematology analyzer (Beckman–Coulter, Brea, CA, USA) in peripheral blood samples from 101 MDS patients and 88 healthy volunteers. MDS blood samples from Tours university hospital (Department of Hematology and Cell Therapy) were collected during the clinical management of patients between May 2015 and July 2017. Exclusion criteria were cytological remission of a hematological malignancy, allogenic bone marrow transplantation and known myeloproliferative/myelodysplastic syndrome, myelofibrosis or iatrogenic dysplasia. The cohort of MDS patients, according to WHO 2016 [23], included nine single lineage dysplasia (MDS-SLD), 28 multilineage dysplasia (MDS-MLD), 8 MDS with ring sideroblasts and single lineage dysplasia (MDS-RS-SLD), 8 MDS-RS with multilineage dysplasia (MDS-RS-MLD), 39 MDS with excess blasts (20 MDS-EB-1 and 19 MDS-EB-2), and 9 MDS with del(5 q). Healthy volunteers had been enrolled in the HEALTHOX and PLASMYC protocols (ClinicalTrials.gov #NCT02789839 and #NCT02809222, respectively). Healthy individuals below 50 years of age were excluded.

Two external testing cohorts were used to perform an independent validation of the MDS-LS, which included 25 healthy controls (without hematological disease) and 22 patients suffering from MDS at diagnosis or during the follow-up of the disease.

### 4.2. Data Collection

The 23 CBC parameters and 103 “research parameters”, containing 5 RUO and 98 CPD parameters (Supplementary Table S4), were collected for all samples. The CBC flags routinely proposed by the manufacturer were systematically collected for each sample.

The CPD parameters include the MN and standard deviation (SD) of volume (V), conductivity (C), and light scatter (S) at different angles for neutrophils (NE), lymphocytes (LY), monocytes (MO), eosinophils (EO), nucleated red blood cells (NRBC), and non-nucleated red blood cells (NNRBC; corresponding to total erythrocytes). The five angles of laser scatter are median angle light scatter (MALS), upper median angle light scatter (UMALS), lower median angle light scatter (LMALS), lower angle light scatter (LALS), and axial light loss (AL2). MALS, UMALS, and LMALS reflect granularity and cellular membrane composition. LALS and AL2 reflect cellular complexity and transparency, respectively [24].

The RUO parameters are low hemoglobin density (LHD), microcytic anemia factor (MAF), platelet distribution width (PDW), early granulated cells (EGC), and white blood cells estimated from the NRBC optical channel (WNOP). LHD could be used to determine iron status and its availability for erythropoiesis [25]. MAF ([hemoglobin x mean corpuscular volume]/100) helps to detect latent iron deficiency [26]. PDW corresponds to the size distribution of the platelet population.

Since MAF is computed from CBC data, it cannot be considered as an unused “research parameter”, and was therefore excluded from our study. Moreover, because the reticulocyte count is not included in basic CBC, parameters derived from reticulocytes were also excluded.

### 4.3. Cross-Validation Strategy to Determine the MDS-LS

The 10 parameters selected to establish the model of interest were those with: (i) the most significant differences between MDS patients and healthy volunteers, (ii) the best contributions to principal component analysis PCA, first axis (dimension 1), and (iii) the best correlations with PCA, first two axes (cos^2^ > 0.6).

The general linear model was obtained by logistic regression to weight parameters, and was validated using a split-sample strategy with 10,000 iterations. For each iteration, 130 subjects were randomly selected in the whole cohort of 189 MDS patients and healthy volunteers. This “learning” group allowed us to build the model. Concomitantly, the remaining 59 subjects of each iteration were included in a “testing” group. When MDS-LS was <0, the subject was classified in the “MDS patient” group by the model, and when MDS-LS was >0, the subject was categorized in the group of “healthy volunteers”. The efficiency of the model was calculated by its ability to correctly classify a sample of the “testing” group by using the model built with the “learning” group. Cumulative distribution function and equation parameters were computed using the “ecdf” function. This mathematical strategy allowed the calculation of the MDS-LS of each sample.

### 4.4. Independent External Testing of the MDS-LS

The MDS-LS was tested in two independent external cohorts of 25 healthy controls and 22 MDS patients. For three MDS patients, two samples were analyzed for a total of 25 CBC. For each patient and control, the 10 critical research parameters were used to calculate the MDS-LS.

### 4.5. Statistical Analyzes

Statistical analyses were performed with R (version 3.5.0) using RStudio software version 1.0.153 (www.rstudio.org). The normal distribution of values was assessed by using the Shapiro–Wilk test (*p* > 0.1) and the homoscedasticity by Levene tests (*p* < 0.05). MN comparisons were computed by using the Wilcoxon and Student tests. PCA were performed using FactoMineR [27]. Logarithmic logistic regression was performed thanks to the “glm” function of the R “stats” package [28].

## 5. Conclusions

Early diagnosis of MDS is of medical interest. This study demonstrates the interest of currently unused “research parameters” of CBC instruments to identify subclinical MDS patients by triggering cytological analysis. The algorithm proposed here, based on logistic regression with 10 WBC parameters, is easily implementable on DxH 800 analyzers. The calculated MDS-LS depends on weighted coefficients, which could be refined by increasing the number of patients tested in prospective and multicentric studies in laboratories equipped with the same instruments. Incorporated into routine clinical use, the MDS-LS could be of clinical interest for early detection of MDS.

## Figures and Tables

**Table 1:** Clinical and Biological Characteristics of the 11 Myelodysplastic Syndrome MDS Patients for Whom MDS-Likelihood Score (LS) Would Have Triggered Blood Smear Review, While Complete Blood Count (CBC) Did Not Generate Any Conventional Flag.

Characteristics	#7	#17	#30	#35	#197	#199	#244	#245	#249	#253	#256	Flag
Age	86	71	82	90	90	68	79	83	89	86	77	
Gender	F	F	M	F	M	M	F	M	M	F	F	
MDS groups	MDS-SLD	MDS-EB-1	MDS-MLD	MDS-RS-SLD	MDS-SLD	MDS-SLD	MDS-RS-MLD	MDS-MLD	MDS-SLD	MDS-MLD	Del(5q)	
Delay from diagnosis (months)	0	1	5	60	1	0	6	NA	0	0	0	
Cytogenetics	normal	normal	normal	normal	tri15	normal	del(11q), tri8	NA	normal	normal	del(5q)	
IPPS-R	1	3	2	2	4	2	4.5	NA	2.5	1	2	
RCC (<120 days)	N	N	N	Y	N	N	Y	NA	Y	N	Y	
Growth factors	N	N	N	N	N	N	N	NA	N	N	N	
Other treatment	N	N	N	N	N	N	N	NA	N	N	N	
RBC (T/L)	3.6	3.2	2.8	2.6	2.3	2.7	3.3	4.4	2.9	3.8	3.5	
Hb (g/L)	104	109	82	81	82	87	100	130	96	114	113	<80
Hct (%)	31.2	32.0	25.1	24.3	23.9	27.3	30.9	38.7	28.4	34.9	34.5	
MCV (fL)	85.8	100.6	90.9	93.1	102.2	101.2	93.0	87.4	97.9	92.6	99.0	>105
MCH (pg/cell)	28.6	34.3	29.9	31.0	35.0	32.3	30.1	29.2	33.0	30.3	32.4	
MCHC (g/dL)	33.3	34.1	32.8	33.3	34.3	31.9	32.4	33.5	33.7	32.7	32.7	>36.0
RDW (%)	14.6	15.0	17.8	16.6	15.8	16.9	18.9	15.4	22.0	14.3	20.1	>22.0
Plt (G/L)	227	146	394	373	111	383	182	104	188	257	447	<100
MPV (fL)	9.3	11.3	8.8	9.4	8.5	7.8	10.7	10.8	8.6	8.6	10.7	<7.0
WBC(G/L)	5.7	7.4	8.4	8.4	3.9	8.2	5.5	5.3	9.6	4.5	2.8	
% NE	67.5	83.4	79.0	74.8	78.7	79.0	61.4	76.5	79.8	46.0	52.6	
% LY	20.9	7.4	15.1	10.0	11.6	10.7	22.7	8.5	6.0	39.9	30.9	
% MO	8.2	8.3	3.4	10.4	6.9	7.1	10.7	12.8	11.2	10.2	9.4	>20.0
% EO	2.4	0.4	1.3	2.5	2.3	2.6	4.2	1.6	1.9	3.5	4.1	
% BA	1.0	0.5	1.2	2.3	0.5	0.6	1.0	0.6	1.1	0.4	3.0	
Abs NE (G/L)	3.8	6.2	6.6	6.3	3.1	6.5	3.4	4.0	7.7	2.1	1.5	<1.5
Abs LY (G/L)	1.2	0.5	1.3	0.8	0.5	0.9	1.3	0.5	0.6	1.8	0.9	>4.0
Abs MO (G/L)	0.5	0.6	0.3	0.9	0.3	0.6	0.6	0.7	1.1	0.5	0.3	>1.5
Abs EO (G/L)	0.1	0.0	0.1	0.2	0.1	0.2	0.2	0.1	0.2	0.2	0.1	>1.5
Abs BA (G/L)	0.1	0.0	0.1	0.2	0.0	0.0	0.1	0.0	0.1	0.0	0.1	>0.3
% NRBC	0.1	0.0	0.0	0.3	0.1	0.2	0.1	0.1	0.5	0.1	0.1	>2.0
Abs NRBC (G/L)	0.01	0.00	0.00	0.02	0.00	0.02	0.01	0.00	0.05	0.01	0.00	
MDS-LS	−6.5	−8.7	−54.4	−10.4	−16.5	−41.9	−23.1	−16.3	−4.0	−2.6	−21.5	<0.0

Abbreviations: F, female; M, male; NA, not applicable; IPSS-R, revised international prognostic scoring system; RCC, red cell concentrates; N, no; Y, yes; RBC, red blood cells; Hb, haemoglobin; Hct, haematocrit; MCV, mean corpuscular volume; MCH, mean corpuscular haemoglobin; MCHC, mean corpuscular haemoglobin concentration; RDW, red cell distribution width; Plt, platelets; MPV, mean platelet volume; WBC, white blood cells; NE, neutrophils; LY, lymphocytes; MO, monocytes; EO, eosinophils; BA, basophils; NRBC, nucleated red blood cells; Abs, absolute value; MDS-LS, MDS-likelihood score.

## Data Availability

No new data were created or analyzed in this study. Data sharing is not applicable to this article.

## Supplementary Materials

The following are available online at https://www.mdpi.com/2072-6694/13/3/389/s1. Table S1: CBC parameters of heathy volunteers and MDS patients. Table S2: research parameters of heathy volunteers and MDS patients. Table S3: MDS-LS and biological characteristics of two external independent cohorts (MDS patients and healthy controls). Table S4: Values of weighting coefficients of the 10 selected parameters and intercept after 10,000 iterations and their median values.
