# Supplementary material for: Automated Early Detection of Myelodysplastic Syndrome within the General Population Using the Research Parameters of Beckman–Coulter DxH 800 Hematology Analyzer
Source: Cancers (Basel). 2021 Jan 21;13(3):389. doi: 10.3390/cancers13030389 (PMC7865695; doi:10.3390/cancers13030389)
Supplement: Supplementary file 1 [file cancers-13-00389-s001.pdf]

Supplementary Table 1. CBC parameters of heathy volunteers and MDS patients

	MDS patients		Healthy volunteers		
	**MN**	**SD**	**MN**	***SD***	***P***
**RBC (T/L)**	2.9	0.6	4.6	0.4	****
**Hb (g/L)**	93.8	18.1	140.2	9.2	****
**Hct (%)**	28.2	5.4	42.0	2.7	****
**MCV (fL)**	99.1	12.3	92.2	4.4	****
**MCH (pg/cell)**	33.0	4.3	30.8	1.6	****
**MCHC (g/dL)**	33.3	1.0	33.4	0.8	
**RDW (%)**	19.1	4.5	13.7	0.7	****
**PLT (G/L)**	154.7	143.8	252.9	48.8	****
**MPV (fL)**	9.6	1.4	8.7	0.8	****
**WBC (G/L)**	4.7	3.8	5.8	1.2	****
**UWBC (G/L)**	4.7	3.8	5.8	1.2	****
**% NE**	54.4	19.8	55.3	7.3	
**% LY**	30.6	18.7	31.9	7.1	
**% MO**	11.2	8.2	8.7	2.1	
**% EO**	2.6	3.4	3.3	2.3	****
**% BA**	1.2	1.3	0.8	0.6	
**Abs NE (G/L)**	2.9	2.9	3.2	0.8	**
**Abs LY (G/L)**	1.1	1.1	1.8	0.6	****
**Abs MO (G/L)**	0.5	0.7	0.5	0.1	**
**Abs EO (G/L)**	0.1	0.1	0.2	0.1	****
**Abs BA (G/L)**	0.05	0.09	0.03	0.05	
**% NRBC**	0.4	0.8	0.04	0.1	****
**Abs NRBC (G/L)**	0.02	0.05	0.002	0.003	****

**: P < 0.01 ; ****: P < 0.0001

Supplementary Table 2. Research parameters of heathy volunteers and MDS patients (MDS-LS parameters are highlighted in grey)

		MDS patients		Healthy volunteers		
		MN	SD	MN	SD	*P*
Neutrophils	MN-V	152,3	14.0	147.7	5.3	**
	SD-V	22	6.4	16.7	1.0	****
	MN-C	143	6.7	144.7	4.6	**
	SD-C	5.9	2.1	4.5	0.5	****
	MN-MALS	132.6	9.3	140.7	4.4	****
	SD-MALS	13.1	4.1	10.3	1.2	****
	MN-UMALS	131.0	10.1	138.8	4.3	****
	SD-UMALS	14.5	4.5	10.8	0.7	****
	MN-LMALS	127.4	10.4	136.0	7.0	****
	SD-LMALS	15.0	3.7	13.0	2.4	****
	MN-LALS	158.4	19.6	165.4	14.5	**
	SD-LALS	31.2	5.8	28.5	4.6	****
	MN-AL2	155.0	8.7	153.7	5.2	
	SD-AL2	14.2	5.1	10.3	1.3	****
Eosinophils	MN-V	160.0	16.7	160.2	5.6	
	SD-V	20.1	7.8	16.7	1.5	***
	MN-C	149.3	12.7	146.5	4.9	
	SD-C	9.1	9.4	4.4	1.8	****
	MN-MALS	194.8	11.2	202.7	4.7	****
	SD-MALS	11.1	4.8	8.4	0.7	****
	MN-UMALS	203.5	12.1	213.1	5.7	****
	SD-UMALS	12.4	5.1	9.2	0.8	****
	MN-LMALS	181.6	11.5	187.7	4.4	****
	SD LMALS	12.5	4.9	10.2	0.9	****
	MN-LALS	157.9	18.1	162.7	8.6	*
	SD-LALS	38.5	8.9	40.2	2.7	*
	MN-AL2	133.4	10.4	126.1	4.4	****
	SD-AL2	12.6	4.6	10.5	1.8	***
NRBC	MN-V	41.0	5.7	46.5	6.3	****
	SD-V	7.5	3.7	6.2	5.7	
	MN-C	58.5	33.8	83.8	36.8	****
	SD-C	37.6	19.2	23.7	21.0	****
	MN-MALS	93.2	14.0	116.0	25.0	****
	SD-MALS	18.1	7.7	17.3	14.1	
	MN-UMALS	69.4	13.6	88.3	26.0	****
	SD-UMALS	18.3	8.6	15.1	13.4	
	MN-LMALS	124.2	19.2	151.8	28.8	****
	SD-LMALS	25.4	10.5	25.0	19.8	
	MN-LALS	110.1	20.6	125.2	26.4	****
	SD-LALS	26.4	11.1	20.7	17.5	*
	MN-AL2	97.2	11.3	99.8	11.2	
	SD-AL2	10.2	4.3	6.8	6.2	****

		MDS patients		Healthy volunteers		
		MN	SD	MN	SD	*P*
Lymphocytes	MN-V	95.1	6.6	88.2	3.1	****
	SD-V	16.3	3.6	14.2	0.9	****
	MN-C	116.1	5.7	115.6	4.1	
	SD-C	9.6	3.9	7.6	1.0	****
	MN-MALS	71.9	7.3	70.6	3.8	
	SD-MALS	18.4	3.5	16.5	0.9	****
	MN-UMALS	73.1	10.8	69.1	6.7	**
	SD-UMALS	21.7	3.4	20.9	1.5	
	MN-LMALS	62.8	5.5	63.3	3.0	
	SD-LMALS	20.3	3.2	18.4	0.8	****
	MN-LALS	41.2	4.4	38.9	2.7	***
	SD-LALS	12.5	2.7	10.6	1.2	****
	MN-AL2	96.3	7.5	92.4	4.7	****
	SD-AL2	13.2	3.8	11.1	0.9	****
EGC	MN-V	167.0	24.3	155.5	12.0	**
	SD-V	30.0	9.5	26.2	10.5	
	MN-C	133.7	7.3	135.9	4.1	**
	SD-C	2.8	1.0	2.1	0.7	****
	MN-MALS	138.1	10.4	147.0	5.5	****
	SD-MALS	7.9	2.4	6.6	2.1	**
	MN-UMALS	144.7	13.5	152.4	7.4	**
	SD-UMALS	12.2	5.4	11.4	3.5	
	MN-LMALS	125.6	11.0	136.2	5.7	****
	SD-LMALS	10.0	5.4	8.8	2.5	
	MN-LALS	119.7	17.6	128.8	12.1	***
	SD-LALS	21.3	6.7	19.8	6.8	
	MN-AL2	165.3	17.0	162.1	10.1	
	SD-AL2	20.6	6.7	15.8	5.4	****
NNRBC	MN-V	122.0	22.8	141.7	12.0	****
	SD-V	50.2	9.1	54.8	5.1	****
	MN-C	108.4	18.7	126.4	15.9	****
	SD-C	38.8	4.1	33.5	3.0	****
	MN-MALS	177.5	17.4	203.6	20.7	****
	SD-MALS	40.2	5.0	42.1	5.0	**
	MN-UMALS	171.0	20.3	200.3	10.6	****
	SD-UMALS	45.0	6.2	49.5	3.5	****
	MN-LMALS	186.1	14.9	211.0	9.0	****
	SD-LMALS	39.6	2.9	36.8	2.1	****
	MN-LALS	71.0	11.2	90.5	6.8	****
	SD-LALS	29.9	6.0	35.4	4.4	****
	MN-AL2	172.3	17.5	183.7	7.2	****
	SD-AL2	33.2	5.5	35.8	2.7	****

		MDS patients		Healthy volunteers		
		MN	SD	MN	SD	*P*
Monocytes	MN-V	184.5	13.7	171.1	5,8	****
	SD-V	23.4	4.6	18.3	2.0	****
	MN-C	123.6	6.2	124.5	4.2	
	SD-C	6.7	4.5	5.2	0.8	*
	MN-MALS	88.2	6.1	87.8	3.1	
	SD-MALS	12.0	3.1	11.1	0.9	
	MN-UMALS	97.9	7.6	96.7	4,5	
	SD-UMALS	13.1	3.9	12.4	1.1	
	MN-LMALS	74.1	6.3	74.4	3.8	
	SD-LMALS	14.5	2.5	13.8	1.3	
	MN-LALS	86.8	14.7	89.3	14.0	
	SD-LALS	24.8	4.9	23.6	4.2	
	MN-AL2	156.8	11.1	154.0	7.1	*
	SD-AL2	15.9	5.6	11.6	1.3	****
RUO	LHD	8.0	8.2	5.8	3.8	
	PDW	18.0	1.2	16.7	0.5	****
	EGC	1.3	3.9	0.1	0.1	****
	WNOP	4.5	3.7	6.4	1.5	****

*: P < 0.05 ; **: P < 0.01 ; ***: P < 0.001 ; ****: P < 0.0001

Supplementary Table 3.

MDS-LS and biological characteristics of two external independent cohorts (MDS patients and healthy controls)

	MDS patients						Healthy controls			
**#**	**Hb (g/L)**	**Plt (G/L)**	**Ne (G/L)**	**PB blasts (%)**	**MDS-LS**	**#**	**Hb (g/L)**	**Plt (G/L)**	**Ne (G/L)**	**MDS-LS**
1	92	19	0.1	3	**-176.4**	26	152	310	3.7	**-11.9**
2	124	79	1.5	7	**-36.6**	27	143	276	6.4	**0.0**
3	113	44	4	0	**-34.7**	28	155	201	6.9	**14.4**
4	127	91	1.2	6	**-41.6**	29	156	156	3.1	**8.9**
5	100	90	0.7	0	**-63.9**	30	134	273	3.9	**11.8**
6	92	130	0.1	0	**-57.2**	31	139	170	3.1	**13.4**
7	89	51	1.6	0	**-77.2**	32	130	201	2.3	**3.7**
8	129	24	0.7	0	**-73.8**	33	146	178	3.5	**9.6**
9	135	23	16	7	**-96.1**	34	124	232	4.1	**13.9**
10	80	385	12.3	0	**-116.0**	35	140	233	5.6	**20.2**
11	102	248	1.9	0	**-21.5**	36	147	247	5.2	**14.8**
12	88	199	4.4	0	**-48.5**	37	154	295	4.9	**11.2**
13	79	125	2.3	0	**-16.3**	38	129	294	5.6	**2.2**
14	103	116	2.8	0	**-5.6**	39	161	181	4.6	**14.5**
15	88	158	1	0	**-88.6**	40	133	218	4.2	**3.4**
16	126	62	3.2	0	**-45.8**	41	136	195	4.7	**-5.8**
17	134	110	1.8	0	**4.3**	42	137	167	3.5	**-27.3**
18	102	36	5.6	0	**0.8**	43	169	170	4.8	**15.6**
19	79	146	2.1	0	**-46.2**	44	137	211	4.9	**2.0**
20	93	43	0.1	7	**-101.8**	45	145	158	2.8	**18.5**
21	71	233	4.5	0	**-37.4**	46	149	226	4.1	**21.1**
22	111	644	2.6	0	**-98.0**	47	133	210	3.3	**-3.0**
23	95	284	4.7	0	**-27.6**	48	140	270	7.1	**8.5**
24	83	276	0.8	1	**-95.5**	49	148	252	5.6	**4.1**
25	93	208	0.6	0	**-69.2**	50	140	313	5.8	**2.4**
**mean**	101	153	3.1	1	**-58.8**	**mean**	143	222	4.6	**7.4**
SD	18.9	140.5	3.7	2.5	41.6	SD	11	47.0	1.3	10.4

Supplementary Table 4. Description of parameters

Parameters	Significance	Technology
**Conventional parameters**		
RBC (T/L)	red blood cells	Coulter principle
Hb (g/L)	hemoglobin	photometry
Hct (%)	hematocrit	calculation
MCV (fL)	mean corpuscular volume	Coulter principle
MCH (pg/cell)	mean corpuscular haemoglobin	calculation
MCHC (g/dL)	mean corpuscular hemoglobin concentration	calculation
RDW (%)	red cell distribution width	calculation
PLT (G/L)	platelets	Coulter principle
MPV (fL)	mean platelet volume	Coulter principle
WBC (G/L)	white blood cells	Coulter principle
UWBC (G/L)	uncorrected white blood cells	Coulter principle
% NE	neutrophils	VCS
% LY	lymphocytes	VCS
% MO	monocytes	VCS
% EO	eosinophils	VCS
% BA	basophils	VCS
% NRBC	nucleated red blood cells	VCS
**Research Use Only (RUO)**		
LHD	low hemoglobin density	calculation
MAF	microcytic anemia factor	calculation
PDW	platelet distribution width	calculation
EGC	early granulated cells	VCS
WNOP	WBC estimate (corrected) from the NRBC optical channel	VCS
**Cell Population Data (CPD)**		
MN-V-X, SD-V-X	mean and SD of volume of X	VCS
MN-C-X, SD-C-X	mean and SD of conductivity of X	VCS
MN-UMALS-X, SD-UMALS-X	mean and SD of upper median angle light scatter of X	VCS
MN-LMALS-X, SD-LMALS-X	mean and SD of low median angle light scatter of X	VCS
MN-LALS-X, SD-LALS-X	mean and SD of low angle light scatter of X	VCS
MN-AL2-X, SD-AL2-X	mean and SD of axial light loss of X	VCS
MN-MALS-X, SD-MALS-X	mean and SD of median angle light scatter of X (UMALS + LMALS)	VCS

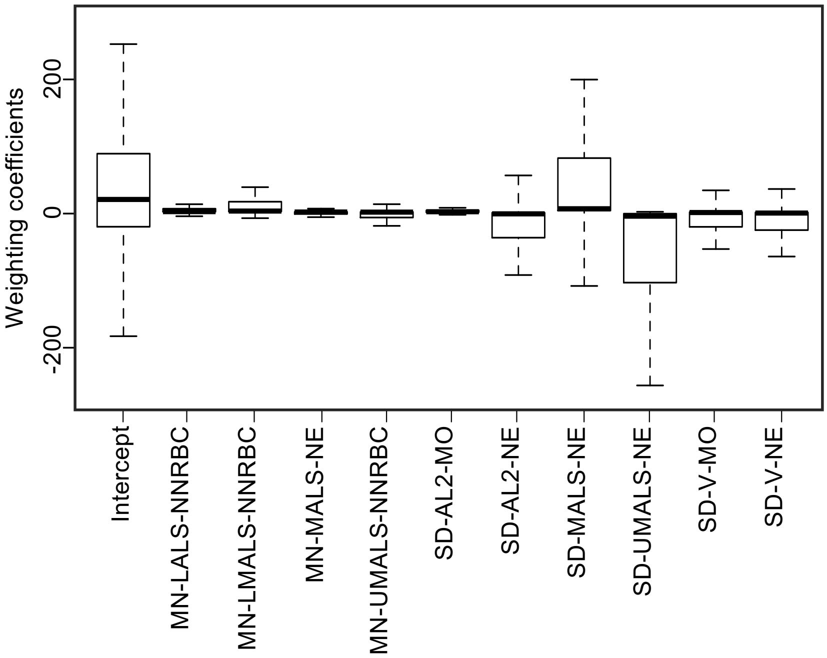

Median values 18.77 0.23 1.14 -0.36 -0.53 0.10 -3.36 4.78 -7.01 -1.19 -2.03

Supplementary Figure 1: Values of weighting coefficients of the 10 selected parameters and intercept after 10,000 iterations
